# Singapore Grouper Iridovirus Induces Glucose Metabolism in Infected Cells by Activation of Mammalian Target of Rapamycin Signaling

**DOI:** 10.3389/fmicb.2022.827818

**Published:** 2022-03-30

**Authors:** Xixi Guo, Qi Zheng, Zanbin Pan, Youhua Huang, Xiaohong Huang, Qiwei Qin

**Affiliations:** ^1^Guangdong Laboratory for Lingnan Modern Agriculture, University Joint Laboratory of Guangdong Province, Hong Kong and Macao Region on Marine Bioresource Conservation and Exploitation, College of Marine Sciences, South China Agricultural University, Guangzhou, China; ^2^Southern Marine Science and Engineering Guangdong Laboratory, Zhuhai, China; ^3^Laboratory for Marine Biology and Biotechnology, Qingdao National Laboratory for Marine Science and Technology, Qingdao, China

**Keywords:** SGIV, glycolysis, HK2, GLUT1, mTOR signaling

## Abstract

Singapore grouper iridovirus (SGIV), a member of the *Iridoviridae* family, is an important marine cultured fish pathogen worldwide. Our previous studies have demonstrated that lipid metabolism was essential for SGIV entry and replication, but the roles of glucose metabolism during SGIV infection still remains largely unknown. In this study, we found that the transcription levels of key enzymes involved in glycolysis were regulated in varying degrees during SGIV infection based on the transcriptomic analysis. Quantitative PCR and western blot analysis also indicated that the expression of both glucose transporters (GLUT1 and GLUT2) and the enzymes of glucose metabolism (hexokinase 2, HK2 and pyruvate dehydrogenase complex, PDHX) were upregulated during SGIV infection *in vivo* or *in vitro*, suggesting that glycolysis might be involved in SGIV infection. Exogenous glucose supplementation promoted the expression of viral genes and infectious virion production, while glutamine had no effect on SGIV infection, indicating that glucose was required for SGIV replication. Consistently, pharmacological inhibition of glycolysis dramatically reduced the protein synthesis of SGIV major capsid protein (MCP) and infectious virion production, and promotion of glycolysis significantly increased SGIV infection. Furthermore, knockdown of HK2, PDHX, or GLUT1 by siRNA decreased the transcription and protein synthesis of SGIV MCP and suppressed viral replication, indicating that those enzymes exerted essential roles in SGIV replication. In addition, inhibition of mTOR activity in SGIV-infected cells effectively reduced the expression of glycolysis key enzymes, including HK2, PDHX, GLUT1, and GLUT2, and finally inhibited SGIV replication, suggesting that mTOR was involved in SGIV-induced glycolysis. Thus, our results not only provided new insights into the mechanism of how SGIV infection affects host cell glycolysis, but also contributed to further understanding of the iridovirus pathogenesis.

## Introduction

Viruses are obligate intracellular parasites that rely on host cellular metabolic system for the energy and macromolecule synthesis required for their replication. In recent years, with the development of technology, studies have focused on examining how virus infection alters host metabolic system. Understanding how virus infection manipulates cellular metabolism is an emerging and critical field for effective vaccine development and virus treatment. Increasing evidence suggested that both DNA viruses and RNA viruses modulated the host cell metabolic profiles after virus infection, including human cytomegalovirus (HCMV), herpes simplex virus 1 (HSV-1), hepatitis C virus (HCV), influenza A virus, human immunodeficiency virus type 1 (HIV-1), Kaposi’s sarcoma-associated herpesvirus (KSHV), vaccinia virus (VACV), Epstein-Barr virus (EBV), infectious spleen and kidney necrosis virus (ISKNV), and so on (Munger et al., 2006, 2008; Diamond et al., 2010; Ritter et al., 2010; Hollenbaugh et al., 2011; Rabinowitz et al., 2011; Roe et al., 2011; Vastag et al., 2011; Delgado et al., 2012; Fontaine et al., 2014; Xiao et al., 2014; Goodwin et al., 2015; Mushtaq et al., 2016; Guo et al., 2019). These reports have revealed numerous dramatic cellular metabolism changes triggered by virus infection. Particularly, the activation of glycolysis has been reported in most of these reports, indicating that glucose is a critical carbon source during virus infection.

Glucose and glutamine are the two main carbon sources for the energetic and biosynthetic needs in cells. In normal cells, glucose is thought to be responsible for cellular ATP generation via glycolysis and the tricarboxylic acid (TCA) cycle. However, in cancer cells, glucose is separated away from the TCA cycle to be used biosynthetically and glutamine serves to anaplerotically replenish the TCA cycle (DeBerardinis et al., 2007, Deberardinis et al., 2008; Wise et al., 2008). For glucose metabolism, exogenous glucose uptake into cells is mediated by glucose transporters (GLUTs), and glucose metabolism was catalyzed by several key enzymes (Adeva-Andany et al., 2016). For example, hexokinases (HKs), which convert glucose to glucose 6-phosphate, is the first and rate-limiting enzyme in glycolytic pathway and the HK2 isoform is a key mediator of aerobic glycolysis (Wolf et al., 2011; Gershon et al., 2013). Pyruvate dehydrogenase complex (PDHX), which provide acetyl-CoA for the TCA cycle, is a multienzyme complex central to aerobic respiration, connecting glycolysis to mitochondrial oxidation of pyruvate (Forsberg et al., 2020).

Iridoviruses are large DNA viruses and result in heavy economic losses in aquaculture industry (Shinmoto et al., 2009). To date, the family Iridoviridae is divided into two subfamilies (*Alphairidovirinae* and *Betairidovirinae*) and categorized into five known genera: *Iridovirus, Chloriridovirus, Lymphocystivirus, Megalocytivirus*, and *Ranavirus* (Chinchar et al., 2017). It has been reported that glucose and glutamine metabolism might play a critical role in the replication of megalocytivirus by producing required energy and metabolites (Fu et al., 2017; Guo et al., 2019). Singapore grouper iridovirus (SGIV) was isolated from diseased grouper (*Epinephelus tauvina*) (Qin et al., 2001, 2003), and characterized as a novel member of the genus *Ranavirus* (Chinchar et al., 2017). The highly lethal and serious systemic disease induced by SGIV infection resulted in great economic losses in aquaculture industry (Gibson-Kueh et al., 2003). The previous studies showed that SGIV infection in host cells evoked non-apoptotic cell death, and mitogen-activated protein kinase (MAPK) signaling pathway involved in SGIV (Huang et al., 2011a,b). During SGIV infection, the level of phosphorylated mammalian target of rapamycin (mTOR) was increased with infection time, suggesting that SGIV inhibited autophagy through decreasing in mTOR activity to some extent (Li et al., 2020). Recently, cellular fatty acid synthesis was demonstrated to exert crucial roles during SGIV infection via regulating virus entry and host immune response (Zheng et al., 2022). Moreover, palmitic acid promoted SGIV replication by suppressing autophagic flux and negatively regulating TANK binding kinase 1 (TBK1)-interferon regulator factor (IRF) 3/7 pathway (Yu et al., 2020). However, the molecular mechanism by which SGIV regulated glucose metabolism still remained largely unknown.

In the present study, the roles of glucose metabolism during SGIV replication was investigated. The expression levels of crucial enzymes of glucose metabolism were examined, and the roles of glycolysis in SGIV infection were investigated using pharmacological inhibitors and siRNA technology. In addition, we also clarified the roles of mTOR in SGIV induced glycolysis. Our results will shed important lights on the mechanism of SGIV pathogenesis, providing new clues to understanding the fish-virus interaction.

## Materials and Methods

### Fish, Cells, and Viruses

Juvenile orange-spotted grouper (weight 30–40 g) were purchased from a local marine fish farm in Hainan Province, China. Fish were maintained in a laboratory recirculating seawater system at 24–28°C and fed twice daily for 2 weeks. Grouper spleen (EAGS) cells were established in our lab (Huang et al., 2009). Grouper head kidney (ELHK) cell used in this study was a new grouper cell line that was established from grouper head kidney (Liu et al., 2021). EAGS and ELHK cells were maintained at 28°C in Leibovitz’s L-15 medium (Gibco, United States) supplemented with 10% fetal bovine serum (Gibco, United States). SGIV was stored in our laboratory (Qin et al., 2003). The virus was propagated in EAGS cells at 28°C and the viral titer was determined by a TCID_50_ assay.

### Reagents

2-Deoxy-D-glucose (2DG) and Dichloroacetate (DCA) were purchased from Sigma-Aldrich. Rapamycin (Rapa, AY-22989) was purchased from Selleckchem. 2DG and DCA were dissolved in L-15 medium to a stock concentration of 500 mM and 400 mM, respectively. Rapamycin was dissolved in DMSO to a stock concentration of 10 mM. These reagents need to be diluted to a working concentration using L-15 medium (2DG and DCA) or DMSO (Rapamycin) just before use. The mouse monoclonal antibody against SGIV major capsid protein (MCP) was prepared and stored in our laboratory. The rabbit polyclonal antibodies against HK2, PDHX, GLUT1, and GLUT2 were purchased from Proteintech (United States). The rabbit monoclonal antibody against β-tubulin was purchased from Abcam.

### Virus Infection and Transcriptomic Analysis

At 5d postinfection (p.i.), three parallel samples of SGIV-infected or mock-infected groupers were harvested. Transcriptomic profiling was performed on an Illumina Hiseq 2500 platform and the analysis process was conducted by Shanghai OE Biotech Co., Ltd. (Shanghai, China).

### Challenge and Samples Collection

A total of 120 healthy Juvenile orange-spotted grouper were randomly divided into two groups, including SGIV- and Mock-infected group (60 fish per group, 20 fish per tank, 3 replicates). The SGIV-infected groups were injected intraperitoneally with 0.1 mL of SGIV (10^5^.^5^ TCID_50_). The Mock-infected groups were injected with 0.1 mL of PBS. Three fish (one fish each duplicate) were randomly selected from SGIV- and Mock-infected group on the 1st, 2nd, 3rd, 5th, and 7th day post-infection. Then the liver, spleen and kidney tissues were obtained for samples preparation.

### Cell Viability

Cell Counting Kit-8 (CCK-8) assay was performed to examine the impact of glucose, glutamine, 2DG and DCA treatment on cell proliferation according to the manufacturer’s instructions. In brief, EAGS and ELHK cells cultured in 96-well plates were incubated with glucose (0, 1.0, and 4.5 g/L), glutamine (0, 2.0 mM), 2DG (0, 0.2, 0.5, 1.0, 2.0, and 5.0 mM) and DCA (0, 0.1, 0.2, 0.5, 0.6, and 1.0 mM) for 24 h. Cells were washed with culture medium three times, and then 100 μL of culture medium supplemented with 10 μL Cell Counting Kit-8 (CCK-8, UE) was added into each well and incubated at 28°C for 4 h. The absorbance was measured in Varioskan™ LUX multimode microplate reader (Thermo Fisher Scientific, United States) at 450 nm.

### Cell Transfection

Cell transfection was carried out using Lipofectamine 2000 reagent (Invitrogen) according to the manufacturer’s protocol. EAGS cells were seeded in 12-well plates at 70–80% confluence for 18–24 h, and cells were transfected with the mixture of 4 μL Lipofectamine 2000 and 100 nM siRNA incubated for 4–6 h. After replacing with fresh normal medium, cells were cultured for further study. The HK2, PDHX, and GLUT1 siRNA was designed by GenePharma. EAGS cells were transfected with HK2, PDHX, and GLUT1 siRNA, respectively, or the same volume of negative control (NC) siRNA for 24 h, and then infected with SGIV for 12 and 24 h.

### Virus Titer Assay

Viral titer was assessed in EAGS cells to determine the effect of glucose, glutamine, 2DG, and DCA on SGIV production, respectively. In brief, EAGS cells were pretreated with glucose, glutamine, 2DG and DCA or vehicle for 2 h, and then the cells were infected with SGIV [at multiplicity of infection (MOI) of 1.0] and collected at 24 h p.i. for virus titer assay. The viral titers of cell lysates were evaluated using the 50% tissue culture infectious dose (TCID_50_) assay (Reed and Muench, 1938). The cytopathic effects (CPEs) were observed under a light microscope (Leica, Germany) every day, and each sample was measured in triplicate.

### RNA Extraction and Real-Time RT-qPCR (qRT-PCR)

EAGS and ELHK cells were infected with SGIV at MOI of 1.0, respectively. The total RNA isolation was performed using the Cell Total RNA Isolation Kit (FOREGENE, China) according the manufacturer’s instructions, and reverse transcription was carried out using ReverTra Ace (TOYOBO, China). Then, qPCR was performed under the ABI Quantstudio™ 5 Real-Time PCR System (Thermo Fisher Scientific, Applied Biosystems, United States). The primers used in this study were listed in Table 1. Reactions of SYBR Green were performed in a 10 μL volume containing 5 μL of 2 × SYBR^®^ Premix Ex Taq™, 0.3 μL of each forward and reverse primer (10 μM), 1 μL of cDNA, and 3.4 μL of water. All experiments were performed in triplicate, and the cycling parameters were chosen according to the manufacturer’s instructions. The expression levels of viral genes and host glycolysis metabolism genes were detected. The relative expression ratio of the selected gene normalized to β-actin was calculated using the 2^–ΔΔ^CT method.

**TABLE 1:** Primers used in this study.

Primer names	Gens names	Sequence (5′–3′)
q-Actin-F	β-Actin	TACGAGCTGCCTGACGGACA
q-Actin-R		GGCTGTGATCTCCTTCTGCA
q-HK2-F	Hexokinase 2	CCCATTGGTTGAGGACTG
q-HK2-R		GTTTCTCGGCTGCTTTGT
q-PDHX-F	Pyruvate dehydrogenase complex	CTGGGTCGTCAAGGGATT
q-PDHX-R		GGGTGACCGAAAGAAGTGT
q-GLUT1-F	Glucose transporter 1	AGGTGTTTGGATTGGAGGTA
q-GLUT1-R		CTGTTGATGAGCAGGAAGC
q-GLUT2-F	Glucose transporter 2	TCTGGGAGCCCTTCTTCATCTGTG
q-GLUT2-R		CGGGGTCATCAACACAGTCTTCAC
q-GLUT4-F	Glucose transporter 4	TGGCGGAGATGAAGGAGGAGAAG
q-GLUT4-R		GCGGTAGAGCGAAGAGCGAAAG
q-MCP-F	MCP	GCACGCTTCTCTCACCTTCA
q-MCP-R		AACGGCAACGGGAGCACTA
q-ICP18-F	ICP18	ATCGGATCTACGTGGTTGG
q-ICP18-R		CCGTCGTCGGTGTCTATTC

### Western Blot Analysis

EAGS and ELHK cells were mock- or SGIV-infected at MOI of 1.0, respectively. After the experimental treatments, cells were lysed and solubilized in 50 μL of Pierce IP Lysis Buffer (Thermo Fisher Scientific), containing protease/phosphatase inhibitor cocktail. Samples were boiled for 5 min after mixing with 5 × loading buffer. The equal of proteins were resolved by 10% SDS-PAGE and then electrophoretically transferred to 0.45 μm TransBlot Turbo PVDF (Minipore). The membranes were blocked with 5% skim milk for 2 h, then incubated with different primary antibodies for 3 h at room temperature or overnight at 4°C. After washing with PBST buffer for 3 times, the membranes were incubated with secondary goat-anti-rabbit or goat-anti-mouse antibody labeled with horseradish per-oxidase. Finally, the immunoreactive bands were visualized with Super ECL Plus Kit (UElandy) according to the manufacturer’s protocol. The following primary antibodies were used: anti-HK2 (1:1,000 dilution), anti-PDHX (1:1,000 dilution), anti-GLUT1 (1:500 dilution), anti-GLUT2 (1:300 dilution), anti-SGIV-MCP (1:3,000 dilution), and anti-β-Tubulin (1:3,000 dilution). Data were normalized to the mean of β-Tubulin expression.

### Determination of Enzyme Activity of Hexokinase 2 and Pyruvate Dehydrogenase Complex in Cells During Singapore Grouper Iridovirus Infection

EAGS cells and ELHK cells were collected at 12 and 24 h after mock- or SGIV infection. HK2 and PDHX activity was measured using Hexokinase Colorimetric Assay Kit (Sigma-Aldrich, MAK091) and Pyruvate Dehydrogenase Activity Assay Kit (Sigma-Aldrich, MAK183) according to the manufacturer’s instructions, respectively. The measured activity was normalized to the total of cells.

### Statistical Analysis

Each sample was assayed in triplicate and results were reported as mean ± standard deviation (SD). Statistical differences between groups were determined by one-way analysis of variance (ANOVA) using SPSS 21.0 software (IBM, United States). Data were considered statistically significant at *p* < 0.05.

## Results

### Singapore Grouper Iridovirus Infection Altered Glucose Metabolism

**FIGURE 1:**
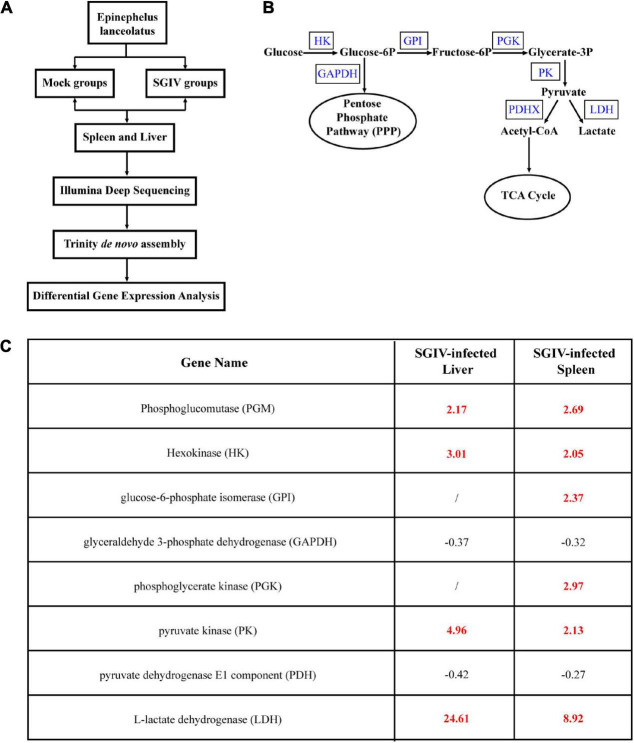
Glucose metabolism is altered during SGIV infection. Grouper were mock- or SGIV- infected and harvested at 5 d for transcriptomic analysis. **(A)** A work flow of transcriptomic design. **(B)** A schema of glucose metabolism, including some glycolytic enzymes (blue typeface showed the enzymes involved in SGIV infection). **(C)** A table visualization of fold changes in levels of glycolytic enzymes profiled during SGIV infection. Bold red values indicate that the mean values are significantly higher in SGIV infected for that comparison, and black values indicate that the values are significantly lower.

To clarify whether glucose metabolism was involved in SGIV infection, we firstly analyzed the transcriptional changes of key enzymes of glucose metabolism pathway based on the transcriptomic data. As shown in Figure 1, the levels of the important glycolytic enzymes were differently expressed in SGIV-infected tissues compared to that in mock-infected tissues. In detail, the transcription levels of phosphoglucomutase (PGM), HK, glucose-6-phosphate isomerase (GPI), phosphoglycerate kinase (PGK), pyruvate kinase (PK) and L-lactate dehydrogenase (LDH) were significantly increased in liver and spleen of SGIV-infected groupers. In contrast, the expressions of the other two glycolytic enzymes, including glyceraldehyde 3-phosphate dehydrogenase (GAPDH) and pyruvate dehydrogenase E1 component (PDH) were decreased (Figure 1). Thus, we speculated that SGIV infection *in vivo* caused the alteration in glucose metabolism.

### Glucose Was Required During Singapore Grouper Iridovirus Infection

**FIGURE 2:**
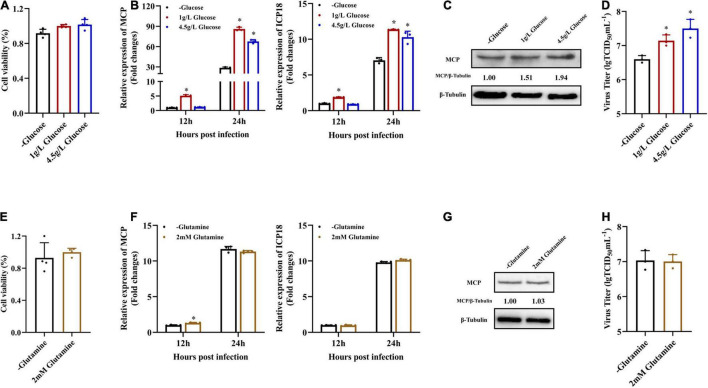
Glucose is necessary for efficient infectious SGIV production. **(A,E)** EAGS cells were cultured in replete medium containing both glucose (1 and 4.5 g/L) and glutamine (2 mM) or medium lacking either glucose or glutamine, and the cell viability was detected by CCK-8 assay at 24 h. EAGS cells were infected with SGIV at MOI of 1.0 and fed replete (1, 4.5 g/L glucose, and 2 mM glutamine), glucose-free, or glutamine-free medium at 2 h. At 12 and 24 h p.i., SGIV was quantified by qRT-PCR **(B,F)**. At 24 h p.i., SGIV was quantified by Western blot **(C,G)** and TCID_50_ **(D,H)**. The data are represented as mean ± SD. The significance level was defined as *p < 0.05.

In order to determine the roles of glucose or glutamine in SGIV replication *in vitro*, the effects of glucose and glutamine deprivation on viral infection were examined. EAGS cells were infected with SGIV at MOI of 1.0 and subsequently fed replete medium containing both glucose (1 or 4.5 g/L), glutamine (2 mM) or medium lacking either glucose or glutamine. Depriving EAGS cells of exogenous glucose or glutamine had no significant impact on cell viability for 24 h (Figures 2A,E). Firstly, the expression of viral MCP in transcription and protein level was determined by qPCR and western blot when SGIV-infected cells was fed with replete medium or medium lacking glucose or glutamine. Deletion of exogenous glucose in the culture medium significantly inhibited the transcription and expression of viral MCP (Figures 2B,C). Furthermore, the virus production was significantly reduced in SGIV-infected exogenous glucose depriving cells. The virus titer in glucose deficient medium was 30 and 50% lower than that in medium containing 1 or 4.5 g/L glucose, respectively (Figure 2D). In contrast, deletion of exogenous glutamine in the culture medium had no significant effects on SGIV replication (Figures 2F–H). These results suggested that glucose was an essential carbon source for SGIV replication.

### Glycolysis Was Activated During Singapore Grouper Iridovirus Infection

**FIGURE 3:**
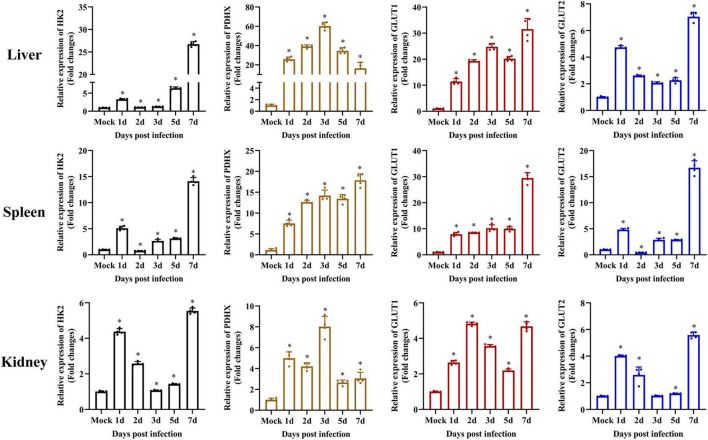
Glycolysis is induced during SGIV infection *in vivo*. The expression of HK2, PDHX, GLUT1, and GLUT2 in grouper tissue (liver, spleen, and kidney). Data were expressed as a ratio to mock expression. The data are represented as mean ± SD. The significance level was defined as *p < 0.05.

It has been reported that exogenous glucose uptake into cells is mediated by glucose transporters (GLUTs), and glucose metabolism is catalyzed by several key enzymes, including HKs and PDHX (Gershon et al., 2013; Adeva-Andany et al., 2016; Forsberg et al., 2020). To determine whether SGIV infection activated glycolysis, the transcription levels of HK2, PDHX, GLUT1, and GLUT2 were firstly examined *in vivo* by qPCR during SGIV infection. The groupers were divided into mock and SGIV infected groups, fish were collected at indicated time points. As shown in Figure 3, HK2, PDHX, GLUT1, and GLUT2 were constitutively expressed in all the analyzed tissues. Among them, HK2, GLUT1, and GLUT2 showed similar expression patterns after SGIV infection, and significantly up-regulated than mock group, then peaked at 7 days in all the analyzed tissues. Interestingly, the expression of PDHX peaked at 3 days in liver and kidney and at 7 days in spleen. These results suggested that SGIV infection promote glycolysis process *in vivo*.

**FIGURE 4:**
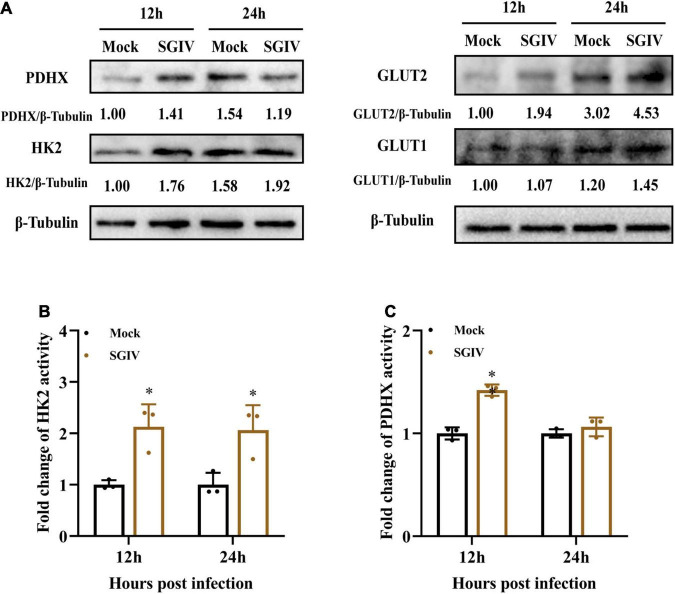
Glycolysis is induced during SGIV infection in EAGS cells. **(A)** Immunoblot analysis of HK2, PDHX, GLUT1, and GLUT2 levels in mock- and SGIV-infected cells. Lysates from cells harvested at 12 and 24 h p.i. were subjected to Western blot analysis using antibodies indicated and β-tubulin was used as the internal control. The values below the lanes indicate the relative intensity of each major band. **(B,C)** The enzyme activity of HK2 and PDHX in SGIV-infected cells compared to mock-infected cells. The data are represented as mean ± SD. The significance level was defined as *p < 0.05.

Furthermore, the protein expressions of those enzymes in mock- and SGIV-infected cells were measured by western blot using specific antibody (Figure 4A). The expressions of HK2, PDHX, GLUT1, and GLUT2 increased in SGIV-infected cells compared to that in mock-infected cells at 12 and 24 h p.i. HK2, GLUT1, and GLUT2 expression increased both at 12 and 24 h p.i. after SGIV infection. Whereas, the expression levels of PDHX first increased at 12 h p.i. and then decreased at 24 h p.i. after SGIV infection. Of note, the expression of GLUT2 increased 1.5-fold in SGIV-infected cells at 24 h compared to that in mock-infected cells. In addition, the activities of HK2 and PDHX was significantly increased in SGIV-infected cells compared to mock-infected cells (Figures 4B,C). Taken together, our results suggested that glycolysis was activated in response to SGIV infection.

### Glycolysis Was Required for Optimal Infectious Singapore Grouper Iridovirus Production

**FIGURE 5:**
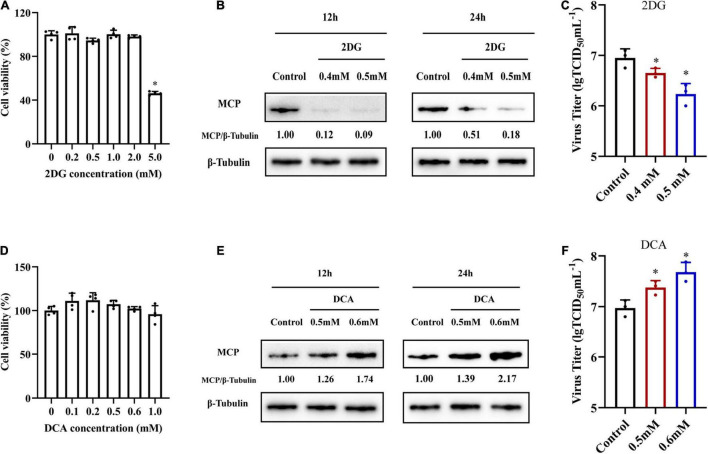
The roles of glycolysis during SGIV replication. **(A,D)** The cytotoxicity of 2DG and DCA on EAGS cells, respectively. **(B,E)** Virus protein level reduced after 2DG treatment and increased after DCA treatment. The level of SGIV-MCP was detected by western blot, and β-tubulin was used as the internal control. **(C,F)** Virus production of SGIV was evaluated. EAGS cells incubated with indicated concentration 2DG and DCA were infected with SGIV and collected at 24 h p.i. Viral titers were determined using the TCID_50_ method. The data are represented as mean ± SD. The significance level was defined as *p < 0.05.

In order to investigate whether glycolysis was involved in SGIV replication, we used specific inhibitors to destroy glycolysis *in vitro*, and then examined the effects of specific inhibitors on SGIV replication. 2DG and DCA are hexokinase inhibitor and pyruvate dehydrogenase kinase (PDK) inhibitor, respectively (Delgado et al., 2010; Kennedy et al., 2019). After determining the cell cytotoxicity of 2DG and DCA on cells (Figures 5A,D), non-toxicity concentration of 2DG (0.4 and 0.5 mM) and DCA (0.5 and 0.6 mM) were used to pretreat cells before SGIV infection. Treatment with 2DG significantly inhibited SGIV replication, evidenced by the decreasing in viral MCP synthesis and production of progeny virus (Figures 5B,C). Moreover, viral protein level and viral titer increased following DCA treatment (Figures 5E,F). The effects of 2DG or DCA on SGIV replication was dose dependent. The similar results also obtained from ELHK cells (Supplementary Figure 1), indicating that glycolysis was involved in the SGIV replication. In addition, we further clarify the effects of inhibition of glutamine metabolism on SGIV replication. EAGS cells were treated with BPTES (an inhibitor of glutaminase) and EGCG (an inhibitor of glutamate dehydrogenase). Consistent with the results shown in Figure 2, the inhibitors of glutamine metabolism have no significant effects on SGIV production (Supplementary Figure 2), indicating that glutamine was not necessary for SGIV replication.

**FIGURE 6:**
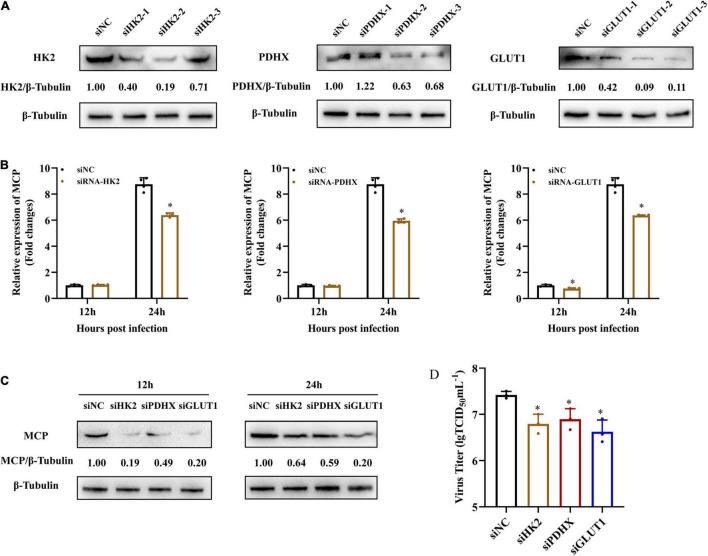
HK2, PDHX, and GLUT1 were essential for SGIV replication. **(A)** The efficacy of different siRNAs targeting grouper HK2, PDHX, and GLUT1 were evaluated by western blotting analysis. Chemically synthesized siRNAs were transfected into grouper cells, and their silencing efficiencies on gene transcription and protein synthesis of HK2, PDHX, and GLUT1 were evaluated by western blotting. **(B–D)** The role of HK2, PDHX, and GLUT1 during SGIV replication. siRNA2-, siRNA2-, and siRNA3- were chosen to evaluate the effect of HK2, PDHX, and GLUT1 silence on SGIV replication, and viral gene transcription and protein synthesis were detected using qPCR, western blotting, and TCID_50_, respectively. Data are expressed as means ± SD. The significance level was defined as *p < 0.05.

To further explore whether grouper HK2, PDHX, and GLUT1 played roles in SGIV replication, we firstly knocked down HK2, PDHX, and GLUT1 in EAGS cells using specific target siRNA, and examined the potent silencing efficiencies of siRNAs on endogenous HK2, PDHX, and GLUT1, respectively. As shown in Figure 6A, the synthesized HK2, PDHX, and GLUT1 protein in siRNA-transfected cells was significantly reduced compared with the negative control siRNA (NC)-transfected cells. Then, siRNA2-HK2, siRNA2-PDHX, and siRNA3-GLUT1 were chosen to evaluate the effects of knock down of HK2, PDHX, and GLUT1 on SGIV replication, respectively. The results showed that knock down of HK2, PDHX, and GLUT1 significantly inhibited SGIV infection, demonstrated by the marked reduction in both transcription and protein expression of SGIV MCP (Figures 6B,C). Moreover, the virus production in siRNA transfected cells were all significant decreased compared with NC-transfected cells (Figure 6D). Thus, our data revealed that HK2, PDHX, and GLUT1 played essential role in SGIV replication.

### Inhibition of Mammalian Target of Rapamycin Signaling Blocked Singapore Grouper Iridovirus-Induced Glycolytic Activation and Attenuated Singapore Grouper Iridovirus Viral Replication

**FIGURE 7:**
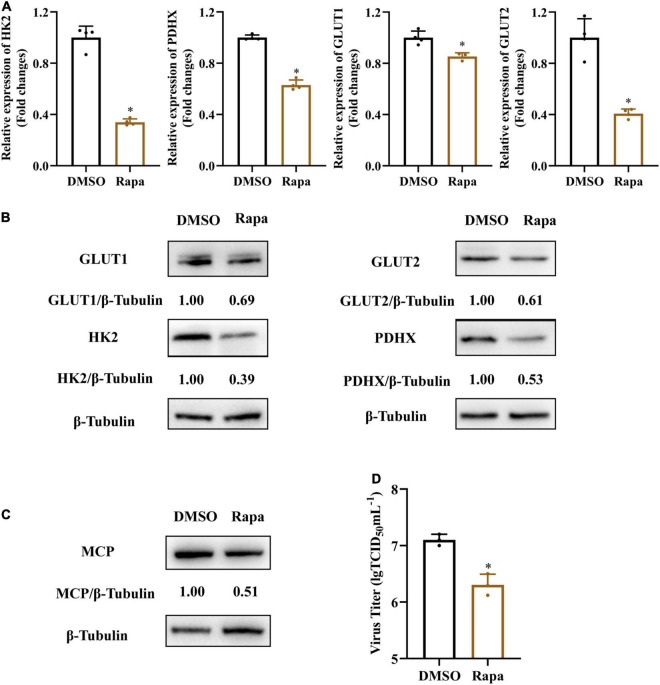
Inhibition of mTOR blocks SGIV-induced glycolytic activation and viral production. Glycolytic enzymes and Gluts expression in SGIV infected EAGS cells with rapamycin treatment for 24 h. **(A)** Total RNA was isolated from the EAGS cells and analyzed by real-time PCR. The expression levels of each gene were normalized to β-actin expression levels and adjusted to the levels in mock-infected EAGS cells. **(B)** The level of glycolytic enzymes and Gluts were detected by western blot, and β-tubulin was used as the internal control. **(C,D)** The impact of mTORC1 on viral protein and, and viral titer using western blotting, and TCID_50_, respectively. Data are expressed as means ± SD. The significance level was defined as *p < 0.05.

Mammalian target of rapamycin (mTOR) signaling plays a critical role in the regulation of energy metabolism, cell growth and proliferation (Mori et al., 2009; Dukhande et al., 2011). To further determine whether mTOR signaling was involved in SGIV-induced activation of glycolysis, we treated SGIV-infected cells with rapamycin, a specific inhibitor of mTOR, and then determined the impact of mTOR inhibition on the expressions of glucose transporters and glycolytic enzymes. As shown in Figures 7A,B, rapamycin treatment dramatically reduced transcription and protein level expression of GLUT1, GLUT2, HK2, PDHX in SGIV-infected cells, indicating that the mTOR activation was critical to regulating SGIV-mediated glycolytic activation in the infected EAGS cells. Furthermore, we also detected the effect of rapamycin on SGIV replication. The results showed that there was a significant decrease in protein level expression and virion production in SGIV-infected cells after rapamycin treatment (Figures 7C,D). Taken together, our results suggested that mTOR signaling was involved in activation of glycolysis induced by SGIV infection.

## Discussion

Viruses hijack host cellular metabolism to provide the building blocks and energy required for successful viral replication. Among them, glucose metabolism is one of an important energy metabolism. During viral infection, the virus can activate glycolysis pathway, which benefit for viral replication. In this study, we examined the alteration of numerous glycolytic enzymes during SGIV infection, and explored the roles of glucose metabolism in SGIV replication. Our transcriptomic results showed that SGIV infection induced the change of numerous glycolytic enzymes, suggesting that glucose metabolism might be involved in the process of SGIV infection. Consistent with transcriptome data, we found the expression levels of key glycolytic enzymes (HK2 and PDHX) and GLUTs were changed *in vitro and in vivo*, with a concurrent increase in enzymes activity of HK2 and PDHX, providing further evidence that SGIV infection can activate glycolysis. The differential expression levels between tissues might be caused by the progress of SGIV infection in different tissues. It has been reported that virus infection could activate glycolysis using different manners. ISKNV infection activates glycolysis through up-regulating the expression of numerous key glycolytic enzymes in infected cells (Guo et al., 2019). EBV LMP1 prompts aerobic glycolysis in infected cells by up-regulation of HK2, which facilitates proliferation by blocking apoptosis (Xiao et al., 2014). HCV activates glycolytic activity by up-regulating the expression of HK2, which directly interact with HCV NS5A protein for viral replication (Ramiere et al., 2014). Dengue Virus (DENV) induces glycolysis for its replication, alone with the up-regulated expression of GLUT1 and HK2 (Fontaine et al., 2015). In addition, Kaposi’s sarcoma herpesvirus (KSHV) infection either induces Warburg effect in the infected cells or employs specific viral microRNAs targeting the key regulators of glucose metabolism and mitochondrial biogenesis in the infected cells to induce glycolytic activity (Delgado et al., 2010, 2012; Yogev et al., 2014). More recently, it was shown that human herpesvirus 6A (HHV-6A) infection increases GLUTs and key glycolytic enzymes expression to prompts glycolysis (Wu et al., 2020). These studies prompted that glucose metabolism may be a common metabolic pathway explored by different viruses to support their replication.

Glucose and glutamine are the two main carbon sources utilized to support the energetic and biosynthetic needs of cells. Viruses utilized glucose or glutamine metabolism to produce required ATP or macromolecule metabolites for virus replication. In our study, we showed that SGIV production was significantly decreased when SGIV-infected cells were deprived of exogenous glucose. In contrast, depriving SGIV-infected cells of exogenous glutamine has no significantly impact on viral yield. The consistent results were obtained when glycolysis was destroyed by specific inhibitors, indicating that exogenous glucose was required for SGIV infection, while glutamine was not. The similar results were reported that Dengue virus required glucose for optimal replication (Fontaine et al., 2015). Differently, Glutamine was required for efficient replication of infectious spleen and kidney necrosis virus (ISKNV) and red-spotted grouper nervous necrosis virus (RGNNV) *in vitro* (Asim et al., 2017; Fu et al., 2017). In addition, white spot syndrome virus (WSSV) induced increased aerobic glycolysis via activation of the PI3K-Akt-mTOR pathway during replications and needed glutamate metabolism to benefit its replication (Su et al., 2014; He et al., 2019). Therefore, activated glycolysis do not represent general host metabolism reprogramming during viral infection, it is more likely that certain viruses have evolved to induce glycolysis pathway to finish their life cycles.

Exogenous glucose across the membranes of cells is mediated by glucose transporters (GLUTs), and glucose metabolism is catalyzed by a number of enzymes, such as HKs, PDHX, lactase dehydrogenase (LDH), and et al. (Gershon et al., 2013; Mueckler and Thorens, 2013; Adeva-Andany et al., 2016; Forsberg et al., 2020). HKs, as a rate-limiting enzyme in glycolytic pathway, had been proved to not only be a new innate immune receptor that activated NOD-like receptor protein 3 (NLRP3) (Wolf et al., 2011, 2016), but also sequester MAVS preventing RIG-I signaling to escape immune response during Hepatitis B virus (HBV) infection (Zhou et al., 2021). In our study, we found that SGIV infection promoted HK2 activity and protein expression, and inhibition of HK2 activity resulted in a significant reduction in SGIV production, suggested that HK2 might be involved in SGIV replication. Of note, PDHX activity and protein expression was promoted at 12 h p.i., suggested that SGIV infection induced PDHX activation and promoted pyruvate into TCA cycle at the early stage of infection. Consistent with the inhibitory effect of siPDHX on SGIV replication, DCA activated PDHX resulted in a significant increase, suggested that PDHX was also essential for SGIV infection. In addition, knockdown of GLUT1 also showed significant inhibition on SGIV replication, suggesting that GLUT1 might enhance the cell capacity to transport glucose and meet increased energy demands following SGIV infection. In addition to regulating glucose transport, GLUT1 can function as a receptor for human T-cell leukemia virus type 1 (HTLV) (Manel et al., 2003). The detailed mechanism of these key enzymes of glycolysis in SGIV infection still needed further investigation.

mTOR is a highly conserved serine/threonine kinase that controls cell growth, cell proliferation and energy metabolism (Duvel et al., 2010; Liu et al., 2015). Our previous studies showed that mTOR pathway participated in autophagy during SGIV infection (Li et al., 2020). Here, inhibition of mTOR activity not only decreases glycolysis in the SGIV-infected cells but also decreased virus replication, suggesting that SGIV-induced glycolysis activation is mainly dependent on mTOR signaling activation. In the other hand, our results also suggested that mTOR exerted a dual role during SGIV infection, regulating glycolysis activation and inhibiting autophagy. Increased literatures revealed that mTOR pathway plays an important role in virus infection. WSSV infection activates PI3K-Akt-mTOR pathway in the infected cells, which is critical for the WSSV-induced aerobic glycolysis (Su et al., 2014). EBV activates AMPK/mTOR/HIF1 pathway to promote glycolysis and induces angiogenesis in nasopharyngeal carcinoma cells (Zhang et al., 2017; Lyu et al., 2018). HHV-6 activates mTOR signaling to promote glycolysis in infected T cells (Wu et al., 2020). Avian reovirus activates the mTORC1/eIF4E/HIF-1α pathway to enhance glycolysis for virus replication (Chi et al., 2018).

In summary, we investigated the roles of glycolysis during SGIV replication. Several genes related to the glucose metabolism were regulated during SGIV infection, and glycolysis inhibition impaired SGIV replication. Furthermore, inhibition of mTOR signaling blocked SGIV-induced glycolytic activation and attenuated SGIV replication, suggesting mTOR signaling involve the glycolytic activation during SGIV replication. Thus, our study provides new insights into understanding the underlying molecular mechanism of glucose metabolism during SGIV infection, which was critical to the development novel therapeutic strategies to restrain viral replication and may provide new insights for preventing viral infection of fish.

## Data Availability Statement

The data presented in the study are deposited in the NCBI public repository under accession number PRJNA804746.

## Ethics Statement

The animal study protocol was approved by the Ethics Committee of Experimental Animals, South China Agricultural University (protocol code 2020g009, 2020.9.8).

## Author Contributions

XG performed the experiments, analyzed the data, and drafted the manuscript. QZ and ZP participated in the qPCR experiments. YH contributed experimental suggestions. QQ and XH designed the experiments and reviewed the manuscript. All authors read and approved the final manuscript.

## Conflict of Interest

The authors declare that the research was conducted in the absence of any commercial or financial relationships that could be construed as a potential conflict of interest.

## Publisher’s Note

All claims expressed in this article are solely those of the authors and do not necessarily represent those of their affiliated organizations, or those of the publisher, the editors and the reviewers. Any product that may be evaluated in this article, or claim that may be made by its manufacturer, is not guaranteed or endorsed by the publisher.

## Supplementary Material

Supplementary Figure 1 The roles of glycolysis during SGIV replication. **(A,D)** The cytotoxicity of 2DG and DCA on ELHK cells, respectively. **(B,E)** Virus protein level reduced after 2DG treatment and increased after DCA treatment. The level of SGIV-MCP was detected by western blot, and β-tubulin was used as the internal control. **(C,F)** Virus production of SGIV was evaluated. ELHK cells incubated with indicated concentration 2DG and DCA were infected with SGIV and collected at 24 h p.i. Viral titers were determined using the TCID50 method. The data are represented as mean ± SD. The significance level was defined as **p* < 0.05.

Supplementary Figure 2 The roles of glutamine metabolism during SGIV replication. **(A)** SGIV transcription level not affected after BPTES and EGCG treatment quantified by qRT-PCR. **(B)** Virus protein level not affected after BPTES and EGCG treatment. The level of SGIV-MCP was detected by western blot, and β-tubulin was used as the internal control. **(C)** Virus production of SGIV was evaluated. EAGS cells incubated with indicated concentration BPTES and EGCG were infected with SGIV and collected at 24 h p.i. Viral titers were determined using the TCID50 method. The data are represented as mean ± SD. The significance level was defined as **p* < 0.05.
