# Supplementary figures and images for: Singapore Grouper Iridovirus Induces Glucose Metabolism in Infected Cells by Activation of Mammalian Target of Rapamycin Signaling
Source: Front Microbiol. 2022 Mar 30;13:827818. doi: 10.3389/fmicb.2022.827818 (PMC9006996; doi:10.3389/fmicb.2022.827818)

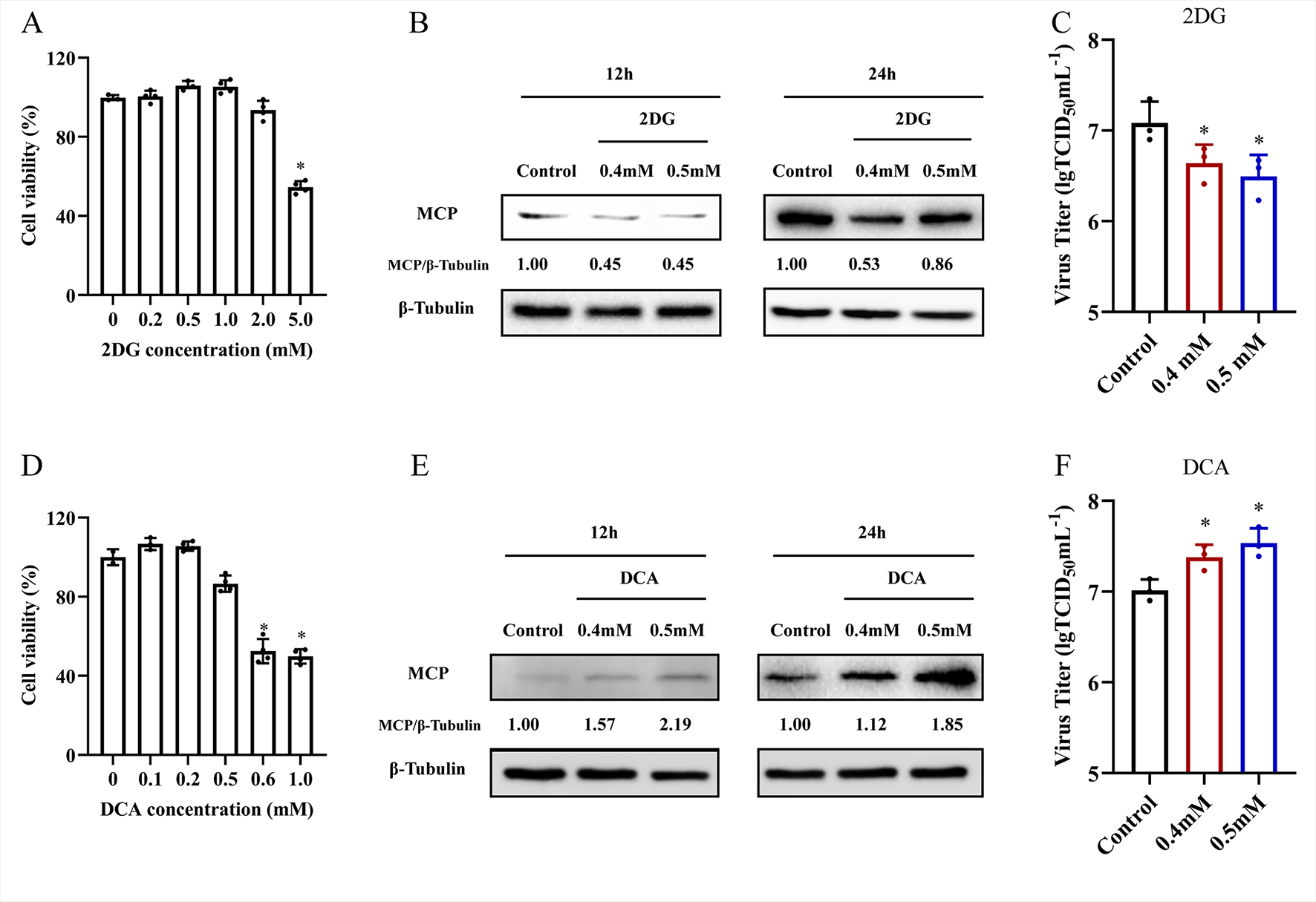

Supplement: Supplementary Figure 1 — The roles of glycolysis during SGIV replication. (A,D) The cytotoxicity of 2DG and DCA on ELHK cells, respectively. (B,E) Virus protein level reduced after 2DG treatment and increased after DCA treatment. The level of SGIV-MCP was detected by western blot, and β-tubulin was used as the internal control. (C,F) Virus production of SGIV was evaluated. ELHK cells incubated with indicated concentration 2DG and DCA were infected with SGIV and collected at 24 h p.i. Viral titers were determined using the TCID50 method. The data are represented as mean ± SD. The significance level was defined as *p < 0.05. [file Image_1.tif]

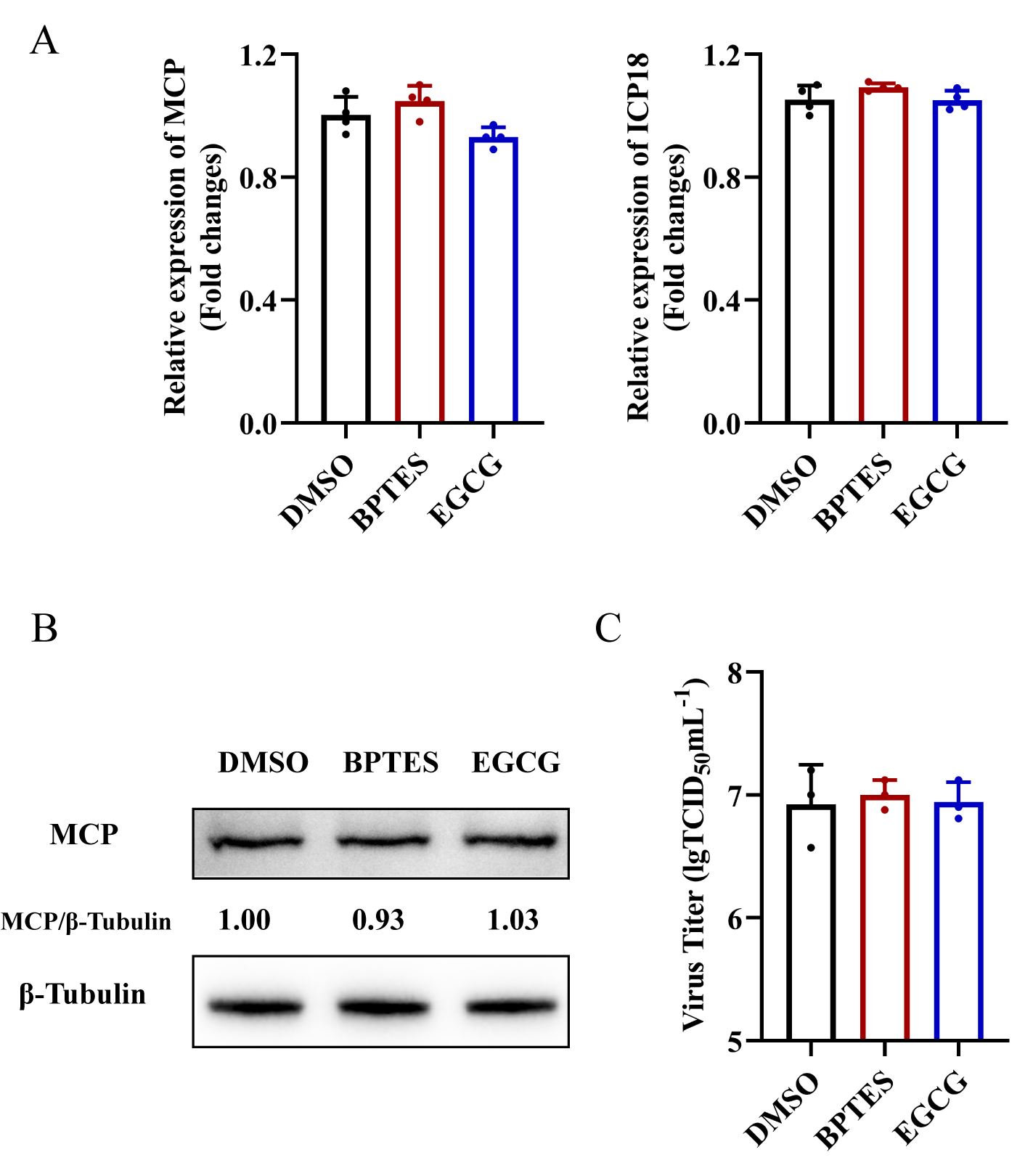

Supplement: Supplementary Figure 2 — The roles of glutamine metabolism during SGIV replication. (A) SGIV transcription level not affected after BPTES and EGCG treatment quantified by qRT-PCR. (B) Virus protein level not affected after BPTES and EGCG treatment. The level of SGIV-MCP was detected by western blot, and β-tubulin was used as the internal control. (C) Virus production of SGIV was evaluated. EAGS cells incubated with indicated concentration BPTES and EGCG were infected with SGIV and collected at 24 h p.i. Viral titers were determined using the TCID50 method. The data are represented as mean ± SD. The significance level was defined as *p < 0.05. [file Image_2.tif]
